# Acclimating Cucumber Plants to Blue Supplemental Light Promotes Growth in Full Sunlight

**DOI:** 10.3389/fpls.2021.782465

**Published:** 2021-11-29

**Authors:** Chenqian Kang, Yuqi Zhang, Ruifeng Cheng, Elias Kaiser, Qichang Yang, Tao Li

**Affiliations:** ^1^Institute of Environment and Sustainable Development in Agriculture, Chinese Academy of Agricultural Sciences, Beijing, China; ^2^Horticulture and Product Physiology, Wageningen University and Research, Wageningen, Netherlands; ^3^Institute of Urban Agriculture, Chinese Academy of Agricultural Sciences, Chengdu, China

**Keywords:** supplemental light, photosynthesis, photoprotection, dynamic light, cucumber, greenhouse

## Abstract

Raising young plants is important for modern greenhouse production. Upon transfer from the raising to the production environment, young plants should maximize light use efficiency while minimizing deleterious effects associated with exposure to high light (HL) intensity. The light spectrum may be used to establish desired traits, but how plants acclimated to a given spectrum respond to HL intensity exposure is less well explored. Cucumber (*Cucumis sativus*) seedlings were grown in a greenhouse in low-intensity sunlight (control; ∼2.7 mol photons m^–2^ day^–1^) and were treated with white, red, blue, or green supplemental light (4.3 mol photons m^–2^ day^–1^) for 10 days. Photosynthetic capacity was highest in leaves treated with blue light, followed by white, red, and green, and was positively correlated with leaf thickness, nitrogen, and chlorophyll concentration. Acclimation to different spectra did not affect the rate of photosynthetic induction, but leaves grown under blue light showed faster induction and relaxation of non-photochemical quenching (NPQ) under alternating HL and LL intensity. Blue-light-acclimated leaves showed reduced photoinhibition after HL intensity exposure, as indicated by a high maximum quantum yield of photosystem II photochemistry (F_*v*_/F_*m*_). Although plants grown under different supplemental light spectra for 10 days had similar shoot biomass, blue-light-grown plants (B-grown plants) showed a more compact morphology with smaller leaf areas and shorter stems. However, after subsequent, week-long exposure to full sunlight (10.7 mol photons m^–2^ day^–1^), B-grown plants showed similar leaf area and 15% higher shoot biomass, compared to plants that had been acclimated to other spectra. The faster growth rate in blue-light-acclimated plants compared to other plants was mainly due to a higher photosynthetic capacity and highly regulated NPQ performance under intermittent high solar light. Acclimation to blue supplemental light can improve light use efficiency and diminish photoinhibition under high solar light exposure, which can benefit plant growth.

## Introduction

Light quality strongly affects the operation and formation of the leaf photosynthetic apparatus and plant growth (Hogewoning et al., 2010; Bugbee, 2016). Wavebands, such as blue, red, or green light, have distinct effects on plant physiological and morphological processes (Claypool and Lieth, 2020; Gao et al., 2020). In modern crop production, plants are often raised by specialized companies before their transfer to the production environment. Specific light spectra may be applied during plant raising to form traits that are desirable for later production, which may occur in greenhouses or the open field. Under full sunlight, plants will experience a longer duration of high light (HL) intensity or a high frequency of HL intensity and low light (LL) intensity transitions, due to variations of the solar angle, cloud cover, overlapping leaves, and greenhouse structures (Li et al., 2014, 2016; Marcelis et al., 2018). Plants need to maximize the efficiency of light energy used for photosynthesis while minimizing deleterious effects associated with exposure to HL intensity. However, how plants acclimated to a given spectrum respond to the dynamic HL intensity exposure is less well explored.

Growing plants under different light spectra involve acclimatory adjustments in the photosynthetic apparatus and plant morphology (Dueck et al., 2016; Landi et al., 2020), but so far most conclusions have been drawn based on experiments in climate chambers (Claypool and Lieth, 2020; Gao et al., 2020). Monochromatic red light often induces the “red light syndrome,” which is characterized by a dysfunctional leaf photosynthetic apparatus and reduced photosynthetic capacity (Hogewoning et al., 2010). Acclimation to blue light, on the other hand, is known to produce “sun-type” plants whose leaves possess high photosynthetic capacity (Savvides et al., 2012). As for plant morphology, increasing the proportion of blue light tends to reduce plant height, whereas monochromatic blue light again increases it, as seen in cucumber (Hernández and Kubota, 2016; Liang et al., 2021), petunia, geranium, calibrachoa, and marigold (Kong et al., 2018). In addition, a green light may penetrate to lower layers of the canopy to benefit photosynthesis in shaded leaves, and increases in the green: blue ratio may serve as a shade signal to promote stem extension and leaf expansion, which may facilitate canopy light interception (Folta and Maruhnich, 2007; Smith et al., 2017). A range of light spectra is currently used in supplemental greenhouse lighting to increase crop growth and to form desired traits. Of course, background sunlight, which exists on top of supplemental light, changes the overall light spectrum to a variable extent and is likely to induce different results compared to results in climate chamber studies (Kaiser et al., 2019; Claypool and Lieth, 2020). Therefore, it is highly relevant to explore acclimation to light spectrum with solar light as a background and to explore how such acclimation affects plant growth and function during exposure to HL intensity.

Plant growth, to a large extent, depends on photosynthetic performance. When exposed to full sunlight, plants often experience dynamic light intensities, such that net photosynthesis rate (*A*; μmol m^–2^ s^–1^) is rarely at a steady state (Kaiser et al., 2015; Li et al., 2016; Marcelis et al., 2018). When light intensity impinging on a shade-adapted leaf increases suddenly, *A* may take 10–30 min to reach a steady state, such that it might not reach a steady state before light intensity decreases again (McAusland and Murchie, 2020). This process, photosynthetic induction, can potentially limit daily CO_2_ fixation by 10–50% (Taylor and Long, 2017; Morales et al., 2018), compared to a hypothetical, instantaneous response of *A* to increases in light intensity. Thus, a promising strategy for increasing light use efficiency in the field is to improve the rate of photosynthetic induction (Taylor and Long, 2017). The stomatal limitation is one of the major determinants of the rate of photosynthetic induction (Sakoda et al., 2021) and is often affected by the growth light spectrum (O’ Carrigan et al., 2014). Therefore, the light spectrum might also affect the rate of photosynthetic induction. A previous study from our lab showed growth-chamber tomato (*Solanum lycopersicum*) plants that were acclimated to different red/blue ratios showed identical photosynthetic induction rates after dark–light transitions (Zhang et al., 2019). However, it is unknown whether acclimation to green and white light affects the photosynthetic induction rate.

When plants are exposed to sunlight, HL intensity can also cause damage to the photosynthetic machinery. The likelihood and severity of deleterious effects of HL intensity exposure are minimized by a set of photoprotective mechanisms. One key process is the controlled dissipation of energy from chlorophyll within PSII, known as non-photochemical quenching (NPQ; Bilger and Björkman, 1990). NPQ may play at least two opposing roles in plant productivity; on the one hand, it reduces photoinhibition and maintains maximal photosynthesis under HL intensity; on the other hand, it momentarily reduces the quantum efficiency of photosynthesis immediately following HL to LL transitions, due to transient overprotection (Murchie and Ruban, 2020). Therefore, a fast speed of formation of NPQ under HL intensity along with a fast relaxation in NPQ under LL is an important target to improve plant productivity under dynamic light (Zhu et al., 2004; Kromdijk et al., 2016; Morales et al., 2018; Foo et al., 2020; Wang et al., 2020). Acclimating plants to different light spectra may modulate NPQ under dynamic light intensity; in tomato and rice (*Oryza sativa*), blue-light-grown leaves (B-grown leaves) displayed faster induction and higher steady-state NPQ, compared to red-light grown leaves (R-grown leaves) (Hamdani et al., 2019; Zhang et al., 2019). Thus, we hypothesize that in greenhouse-grown plants, acclimation to blue supplemental light could enhance NPQ formation and relaxation and biomass production after HL intensity exposure, relative to acclimation to extra red light.

The aim of this study is to investigate how acclimation to a given spectrum in LL “prepares” plants for HL intensity exposure. Photosynthetic and photoprotective responses to stable and dynamically changing HL intensity, and the consequences of this acclimation for plant growth under full sunlight were investigated.

## Materials and Methods

### Plant Materials and Growth Conditions

Cucumber (*Cucumis sativus* cv. Xiamei No. 2) seeds (130–150 seeds per batch of experiment) were sown in Rockwool plugs (Grodan, Roermond, Netherlands) and germinated in a growth chamber at a photoperiod of 16 h, photosynthetic photon flux density (PPFD) of 100 μmol m^–2^ s^–1^ provided by white light-emitting diode (LEDs), and at an ambient CO_2_ partial pressure, the temperature of 23 ± 1°C, and relative humidity of 70 ± 10%. When plants unfolded the 1st true leaf (2 weeks after sowing), seedlings were transplanted to Rockwool cubes (10 cm × 10 cm × 6.5 cm, Grodan, Roermond, Netherlands); ∼100 seedlings per batch of experiment, and transferred to a Venlo-type glasshouse (Beijing, China, 40°N, 116°E) for experimental treatment (refer to Table 1 for environmental conditions).

**TABLE 1:** Growth conditions during four batches of the experiment.

Batch number	Duration	Photoperiod (h)	DLI of solar light (mol m^–2^ day^–1^)	DLI of solar light and LED (mol m^–2^ day^–1^)	Average T (day/night) (°C)	Average RH (day/night) (%)	[CO_2_] range (μmol mol^–1^)
1	June 28 to July 7	14.9	3.5	7.8	26/23	73/83	400–430
2	July 22 to July 31	14.5	2.8	7.1	27/24	76/86	
3	August 12 to August 21	13.6	2.1	6.4	28/25	72/86	
4	September 11 to September 20	12.4	2.4	6.7	25/21	56/62	
4 (FSL)	September 21 to September 27	12.4	10.7	-	24/20	67/79	

*DLI, daily light integral; T, temperature; RH, relative humidity; [CO_2_], CO_2_ partial pressure; FSL, full solar light treatment.*

A white sunscreen (Harmony 6145, transmission of 39%, Ludvig Svensson, Kinna, Sweden) was placed at the top of the greenhouse (below the gutter) to reduce the incoming sunlight intensity during the experiment unless specified. Five cultivation tables with aluminum alloy frames (200 cm L × 120 cm W × 180 cm H) were arranged from east to west with 80 cm between tables. The top and sides of each frame were covered by white sunscreen for further shading. Five light treatments were arranged randomly, one per cultivation table: control (C, without supplemental light on), white (W), red (R), blue (B), and green (G). All treatments received identical amounts of background solar light (Table 1). LED lamps (ZWS01D-LED120-180, Panan Greenlight, Jinhua, China) were installed 70 cm above the cultivation table and were turned on from 08:00 to 18:00, at an intensity of 120 μmol m^–2^ s^–1^ measured 20 cm above the cultivation table. Peak wavelengths of R, B, and G LEDs were 656, 451, and 519 nm, respectively (Supplementary Figure 1). The LED lamp intensity was monitored with a spectroradiometer (Avaspec-2048CL, Avates, Apeldoorn, Netherlands). PPFD of solar light inside the greenhouse (Table 1) was recorded continuously with a line sensor (Licor191R, Li-COR, Lincoln, NE, United States).

The greenhouse experiment was performed from June 28, 2020 to September 27, 2020, during which four batches of plants were grown in succession (Table 1). Daily light integral (DLI) from sunlight was ∼2–3.5 mol photons m^–2^ d^–1^, depending on the batch (Table 1). Per batch, treatments were re-arranged randomly, and 12–20 plants per treatment and batch were grown for 10 days. Additionally, in the fourth batch, after 10 days of treatment, sunscreens were removed and plants from all treatments were exposed to full solar light, without supplemental light, for 1 week (refer to Table 1 for environmental conditions during the complete experiment and Supplementary Figure 2 for daily environmental conditions during full solar light exposure).

Plants were irrigated with modified Hoagland nutrient solution (pH = 5.8, EC = 2.0 dS m^–1^) regularly, and plants within treatments were rotated randomly every day. Greenhouse temperature, relative humidity, and CO_2_ partial pressure were continuously recorded with a climate sensor (TR-76Ui, T&D Co. Ltd., Nagano, Tokyo, Japan; Table 1).

### Measurements

The number of traits pertaining to plant growth and photosynthetic acclimation was quantified after 10 days of treatment and after 7 days of subsequent exposure to full sunlight. Unless specified, all leaf-level measurements were conducted on the youngest fully expanded leaf (∼15-cm width). Measurements conducted in each experimental batch are detailed in Table 2. Three to six biological replicates (plants) were performed per treatment per experimental batch.

**TABLE 2:** Measurements conducted in each batch of the experiment.

Measurement	Batch number			
	1	2	3	4
Leaf biochemical components	√		√	√
Leaf optical properties			√	
Leaf cross-section microscopy			√	
Stomatal morphological traits			√	
Plant growth analysis	√	√	√	√
Gas exchange and chlorophyll fluorescence measurement using LI-6400XT		√	√	√
Chlorophyll fluorescence imaging			√	
Photosynthesis and growth analysis after full solar light exposure				√

#### Leaf Biochemical Components

Leaf discs (4 × 1.0 cm^2^) were stored for 36 h in darkness in 8 ml 95% ethanol at 4°C. The absorbance of the extract was measured at 470, 649, and 665 nm, using a spectrophotometer (UV-1800, Shimadzu, Kyoto, Japan). Chlorophyll and carotenoid contents were calculated according to Wellburn (1994). Dry leaf samples (0.2 g) were ground to powder and used to measure total nitrogen (N) and carbon (C) content with a C/N analyzer (vario PYRO cube, Isoprime, Cheadle Hulme, United Kingdom).

#### Leaf Optical Properties

Leaf reflectance (Rf) and transmittance (Tr) were measured with a spectrophotometer (USB2000 +, Ocean Optics, Dunedin, FL, United States) with two integrating spheres (FOIS-1, ISP-REF, Ocean Optics, Dunedin, FL, United States). Leaf absorptance (Ab) was calculated as Ab = 1 − (Rf + Tr).

#### Stomatal Morphology

The silicon rubber impression technique (Savvides et al., 2012) was used to determine stomatal traits of both leaf surfaces. As a stomatal impression needed to be made on flat leaves, we used the leaves under the cultivation irradiance that had been detached immediately before (within 5 s from detachment to the application of silicon rubber on the table). Epidermal impressions were observed with an optical microscope (XSP-13 CC, Shanghai Caikon Optical Instruments, Shanghai, China) that was equipped with a digital camera (CK-300, Shanghai Caikon Optical Instruments, Shanghai, China). Under a magnification of × 400, five visual fields (19678.08 μm^2^) were randomly selected per sample, and numbers of stomatal and epidermal cells were counted. About 20 stomata per visual field were picked randomly to measure stomatal length and width, and pore length and aperture; from these, pore and stomatal area were calculated under the assumption that these were elliptical. Stomatal density, stomatal index, and pore area per leaf area were calculated according to Savvides et al. (2012).

#### Leaf Cross-Section Microscopy

Leaf segments (2 × 1 cm) of the youngest fully expanded leaves were cut and fixed in a formaldehyde-based fixative formalin-aceto-alcohol (FAA) for at least 24 h. After that, leaf segments were dehydrated and embedded in paraffin, and sectioned with a microtome (RM2016, Leica Microsystems, Shanghai, China). The sections were stained with safranin along with Fast Green and were examined using a microscope (BX53, Olympus Optical Co. Ltd., Tokyo, Japan).

#### Gas Exchange and Chlorophyll Fluorescence

Steady-state and dynamic leaf photosynthetic gas exchange was measured using the LI-6400 XT photosynthesis system (LI-COR Biosciences, Lincoln, NE, United States) equipped with the leaf chamber fluorometer (LI-COR Part No. 6400-40, enclosed leaf area: 2 cm^2^). During measurements, CO_2_ partial pressure was 400 μbar, leaf temperature was approximately 25°C, leaf vapor pressure deficit was 0.7–1.0 kPa, and air flow rate through the system was 500 μmol s^–1^. PPFD was provided by a mixture of red (90%; 635 nm) and blue (10%; 465 nm) LEDs in the leaf chamber.

##### Light Response Curves of Leaf Photosynthesis

Leaves were firstly adapted to 1500 μmol m^–2^ s^–1^ PPFD, until *A* was stable, after which they were exposed to 1,000, 800, 600, 400, 200, 150, 100, 50, and 0 μmol m^–2^ s^–1^ PPFDs. When *A* reached steady-state at each PPFD (3–5 min), *A*, g_*s*_, and C_*i*_ were logged continuously (every 5 s) for 1 min, and averaged values were used. Light response curves were fitted to a non-rectangular hyperbolic function (Cannell and Thornley, 1998), and the parameters maximum net photosynthesis rate (*A*_*max*_), dark respiration rate (R_*dark*_), and apparent quantum yield (α) were derived.

##### Dynamic Photosynthetic Responses to Step Changes in Irradiance

To evaluate gas exchange and chlorophyll fluorescence responses to a step change in PPFD, plants were adapted in a dark room for ∼30 min. After that, selected leaflets were placed in the LI-6400 XT cuvette, and minimal (F_0_) and maximal (F_*m*_) chlorophyll fluorescence were recorded to determine the maximum quantum efficiency of PSII photochemistry (F_*v*_/F_*m*_). PPFD was then increased to 50 μmol m^–2^ s^–1^, and leaves were adapted at this PPFD until *A* and stomatal conductance (g_*s*_) were at a steady state (approximately 30 min). After that, leaves were subjected to six cycles of 2 min LL (50 μmol m^–2^ s^–1^) followed by 5 min of HL intensity (1,000 μmol m^–2^ s^–1^) each, for a total of 42 min. In the end, leaves were subjected to 10 min of darkness. Gas exchange was logged once per second and transient *A*, g_*s*_, and C_*i*_ responses were averaged over five data points to reduce measurement noise, using a moving average filter. Photosynthetic induction state was calculated, and the following parameters: (1) photosynthetic induction state 60 and 300 s after illumination (IS_60_ and IS_300_); and (2) *A*_300_, integrated *A* during the first 300 s of photosynthetic induction.

Chlorophyll fluorescence was measured once per minute, using the multiphase flash (MPF) routine. “Steady-state” fluorescence yield (F_*s*_) and maximum fluorescence (F_*m*_′) in the light were determined. MPF settings were as follows: measuring intensity was 1 μmol m^–2^ s^–1^, the maximum flash intensity was 8,000 μmol m^–2^ s^–1^, flash intensity reduced by 60% during the 2nd phase of the MPF, and the duration of the three flash phases were 0.3, 0.6, and 0.3 s, respectively. Photosystem II operating efficiency (Φ_*PSII*_) was calculated as Φ_*PSII*_ = (F_*m*_′-F_*s*_)/F_*m*_′, and NPQ was calculated as NPQ = F_*m*_/F_*m*_′-1 (Baker, 2008). Two components of NPQ, photoinhibitory quenching (qI) and energy-dependent quenching (qE), were calculated: qE and qI before the dark relaxation period were calculated according to Liu and Last (2015) as qI = (F_*m*_-F_*m*_″)/F_*m*_″, and qE = F_*m*_/F_*m*_′-F_*m*_/F_*m*_″, where F_*m*_′ is maximum fluorescence immediately before dark relaxation, and F_*m*_″ is maximum fluorescence after 10 min of dark relaxation.

##### Chlorophyll Fluorescence Imaging After High Light Stress

To evaluate leaf photoprotective capacity, F_*v*_/F_*m*_ before and after HL treatment was measured using the Imaging-PAM Chlorophyll Fluorescence System (MAXI-PAM, Heinz Walz GmbH, Effeltrich, Germany). Plants were first dark-adapted for 30 min, and F_0_ and F_*m*_ of the youngest fully developed leaves (target leaves) were recorded to determine F_*v*_/F_*m*_. Then, plants were moved to the greenhouse (low-intensity sunlight) for ∼60 min to reach a low-light adapted state. Plants were then moved to a customized facility with white LEDs and exposed to HL of 1,100 μmol m^–2^ s^–1^ for 30 min. Later, plants were dark-adapted for 30 min, and F_*v*_/F_*m*_ was determined once more.

##### Leaf Photosynthetic Capacity After Full Solar Light Exposure

To follow photosynthetic capacity in single leaves after full solar light exposure, the 2nd true leaf, counted from the bottom of the plant, was used for measurements. At 0, 2, 4, and 6 days after exposing plants to full sunlight, leaves were exposed to 1,000 μmol m^–2^ s^–1^ PPFD (close to the light saturation point), using the LI-6400 XT. When *A* was stable, gas exchange parameters were logged continuously (every 5 s) for 1 min, and averages of 12 values were used.

#### Growth Analysis

Growth analysis was conducted after 10 days of treatment and after 1 week of full solar light exposure. Leaf area was measured using a leaf area meter (LI-3100C, LI_COR Biosciences, Lincoln, NE, United States). Leaves and stems were dried in a ventilated oven (DHG-9070A, Shanghai Jinghong, Shanghai, China) at 80°C for at least 72 h. Leaf mass area (LMA) was calculated as leaf dry weight/leaf area.

### Data Analysis

Statistical analysis was performed using IBM SPSS version 23 (IBM Corp., Armonk, NY, United States). For measurements conducted in several batches of experiments (Table 2), the average value per batch was treated as one statistical replicate. For measurements conducted only in one batch of the experiment (Table 2), each plant was treated as one biological replicate. One-way ANOVA was performed followed by Duncan’s test at 95% CI. Data were plotted using SigmaPlot 12.5 (Systat Software, Inc., San Jose, CA, United States).

## Results

### Leaf Thickness, Pigmentation, and Stomatal Traits

Leaf mass area (LMA) in plants grown under supplemental light increased by 27–55% (Table 3), compared to control plants. Growth under blue light produced the thickest leaves, as these showed ∼12% greater LMA than W-, R-, and G-grown leaves (Table 3); these leaves were visibly thicker (Supplementary Figure 3). All supplemental light treatments increased chlorophyll (Chl *a* + *b*), carotenoid (cars), and nitrogen and carbon concentrations (Table 3) compared to control. Similar to LMA, the largest concentrations of these components were found under B, followed by W, R, and G treatments (Table 3). The Chl *a*:*b* ratio was unaffected by treatments (Table 3). Leaf light absorption was 5–7% higher in leaves grown under supplemental light compared to control, without differences among supplemental light treatments (Table 3 and Supplementary Figure 4).

**TABLE 3:** Leaf biochemical, photosynthetic, and morphological traits of cucumber plants grown under different supplemental light spectra.

Parameter	Light quality					*p*-value
	Control	White	Red	Blue	Green	
Chl *a* + *b*(mg m^–2^)	24229d	31930*ab*	31019*bc*	34227a	28631c	<0.001
Chl *a:b*	2.720.08	2.790.05	2.780.01	2.720.08	2.710.03	0.609
Carotenoid (mg m^–2^)	33.85.2c	46.53.3a	45.02.0a	48.94.0a	39.43.1b	<0.001
N (g m^–2^)	0.820.06d	1.130.09b	1.030.05c	1.350.13a	1.020.09c	<0.001
C (g m^–2^)	5.260.34d	7.260.26b	7.060.28b	8.180.46a	6.740.42c	<0.001
*A*_max_ (μmol m^–2^ s^–1^)	12.60.7d	21.71.3b	17.90.7c	26.70.9a	16.90.8c	<0.001
α (μmol m^–2^ s^–1^)	0.0650.003	0.0670.001	0.0650.002	0.0690.003	0.0630.002	0.545
R_dark_ (μmol m^–2^ s^–1^)	1.400.10c	1.930.15*ab*	1.790.08b	2.150.08a	1.620.14*bc*	0.005
LMA (g cm^–2^)	12.81.0d	17.70.8b	17.00.8*bc*	19.91.5a	16.31.2c	<0.001
Light absorption (%)	81.80.9b	87.50.4a	87.80.2a	86.70.1a	86.30.2a	<0.001

*Control, shade solar light; Chl a + b, total chlorophyll a and b contents; Chl a:b, chlorophyll a and b ratio; N, total leaf nitrogen content; C, total leaf carbon content; A_max_, maximum net photosynthesis rate; α, apparent quantum yield; R_dark_, dark respiration rate; LMA, leaf mass area. Mean value ± SEM of three experimental batches is shown (n = 3) except in light absorption, the mean value ± SEM of six biological replicates in one experiment is shown. The p-values of treatment effect are shown, and different letters indicate significant treatment effect.*

Stomatal traits were measured in one batch of the experiment. Growth under supplemental light increased stomatal density on both leaf surfaces, although this was not significant on the adaxial side (Table 4 and Supplementary Figure 5). B significantly increased the single stomatal area, as B-grown leaves had the longest and widest stomata. Total pore area per leaf area on the abaxial side was largely increased by supplemental light, particularly for B-grown leaves, where the total pore area per leaf area was ∼80% greater than that in control leaves (Table 4).

**TABLE 4:** Stomatal traits on the adaxial and abaxial surfaces of cucumber leaves.

	Light quality					*p*-value
	Control	White	Red	Blue	Green	
**Stomatal density (no.mm^–2^)**						
Adaxial	27012	51024	44275	47344	44970	0.059
Abaxial	35839b	69522a	57044a	63569a	56045a	0.005
**Stomatal index (−)**						
Adaxial	0.120	0.130.01	0.110.01	0.160	0.140.02	0.093
Abaxial	0.190	0.190.02	0.330.18	0.220.01	0.180.02	0.712
**Stomatal area (μm^2^)**						
Adaxial	1647b	16412b	15312b	2062a	1745b	0.010
Abaxial	1664b	1743b	1823b	20911a	1761b	0.004
**Stomatal length (μm)**						
Adaxial	19.30.5	17.61.1	17.11.4	19.80.2	18.60.4	0.241
Abaxial	18.70.2*ab*	18.10.4b	18.80.2*ab*	19.50.3a	18.30.1b	0.032
**Stomatal width (μm)**						
Adaxial	10.90.2c	11.90.2b	11.40.1*bc*	13.30.3a	11.90.4b	0.001
Abaxial	11.30.1c	12.20.3*bc*	12.30.2b	13.50.5a	12.20.1*bc*	0.005
**Pore aperture (μm)**						
Adaxial	2.80.2	3.50.5	3.30.3	4.40.4	3.80.6	0.184
Abaxial	3.80.1	4.20.4	4.10.1	5.10.3	4.30.4	0.085
**Pore length (μm)**						
Adaxial	10.80.6	9.00.8	9.51.1	9.60.2	10.70.5	0.360
Abaxial	11.10.6	10.00.3	12.20.4	11.00.2	10.60.6	0.063
**Pore area per leaf area (μm^2^ mm^–2^)**						
Adaxial	6360234	131932951	106831634	159162167	150914883	0.194
Abaxial	9087468c	232513406*ab*	223251216*ab*	281123477a	157121051*bc*	0.001

*Cucumber plants were grown for 10 days under different supplemental light spectra: Control, shade solar light; White, supplemental white light; Red, supplemental red light; Blue, supplemental blue light; and Green, supplemental green light. Mean value ± SEM of three biological replicates in one experimental batch is shown (n = 3). The p-values of treatment effect are shown, and different letters indicate significant treatment effect.*

### Steady-State Photosynthesis Traits

**FIGURE 1:**
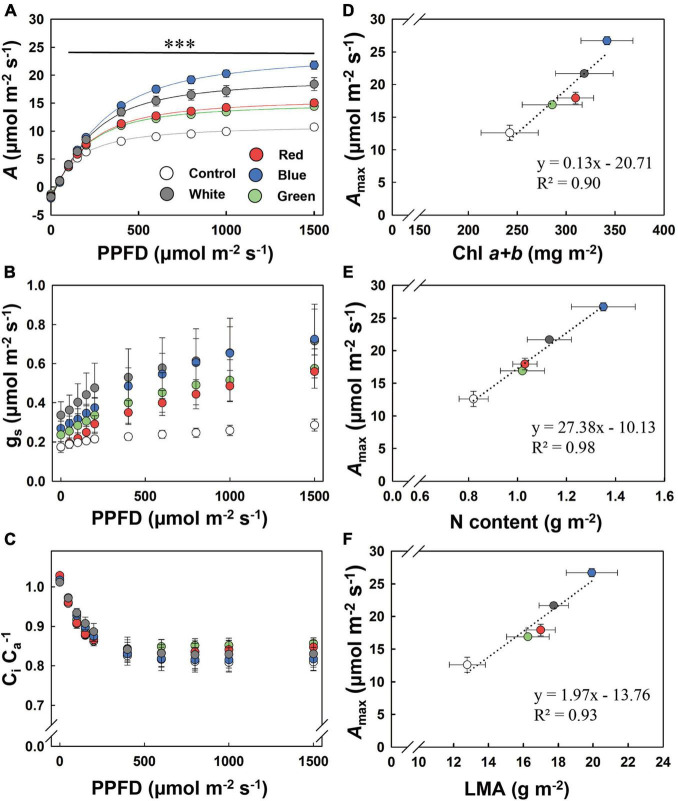
Response of steady-state net leaf photosynthesis (*A*; **A**), stomatal conductance (g_*s*_; **B**), and leaf internal CO_2_ partial pressure relative to that of the air (C_*i*_C_*a*_^–1^; **C**) to photosynthetic photon flux density (PPFD), and relationship of maximum photosynthesis rate (*A*_*max*_) to chlorophyll *a* and *b* contents (Chl *a* + *b*; **D**), N content per leaf area (N; **E**), and leaf mass area (LMA; **F**). Control, shade solar light. Mean value ± SEM of 3–4 experimental batches is shown (*n* = 3–4), with four to six replicate plants per experimental batch. The asterisks in **(A)** indicate significant differences between treatments, ^***^p < 0.001. The dotted line in (**D–F)** represents a significant linear regression, with equations and *R*^2^ coefficients shown.

Steady-state *A* increased remarkably by acclimation to supplemental light, as shown by *A* in response to PPFD (*A*/PPFD curve, Figure 1A). When PPFD was above 150 μmol m^–2^ s^–1^, B-grown leaves displayed the highest *A*, followed by W-, R-, and G-grown leaves, resulting in an *A*_*max*_ that was more than twice as high in B leaves compared to control leaves (Table 3). Blue light accounted for 72%, 26%, 17%, and 10% of PAR in B, W, G, and R supplemental light treatments, respectively. We found a positive correlation between blue light proportion during growth and *A*_*max*_ (Supplementary Figure 6). In addition, stomatal conductance (g_*s*_) across a range of PPFD tended to be the highest in B-grown leaves (Figure 1B). The ratio of leaf internal to ambient CO_2_ partial pressure (C_*i*_ C_*a*_^–1^) was unaffected by treatments (Figure 1C). Across treatments, *A*_*max*_ correlated positively with Chl *a* + *b*, N content, and LMA (Figures 1D–F). Dark respiration (R_*dark*_) was significantly higher under supplemental light treatments and was highest under B, followed by W, R, and G (Table 3). Photosynthetic quantum yield (α) was not affected (Table 3).

### Dynamic Responses of Leaf Photosynthesis During Changes in Irradiance Intensity

**FIGURE 2:**
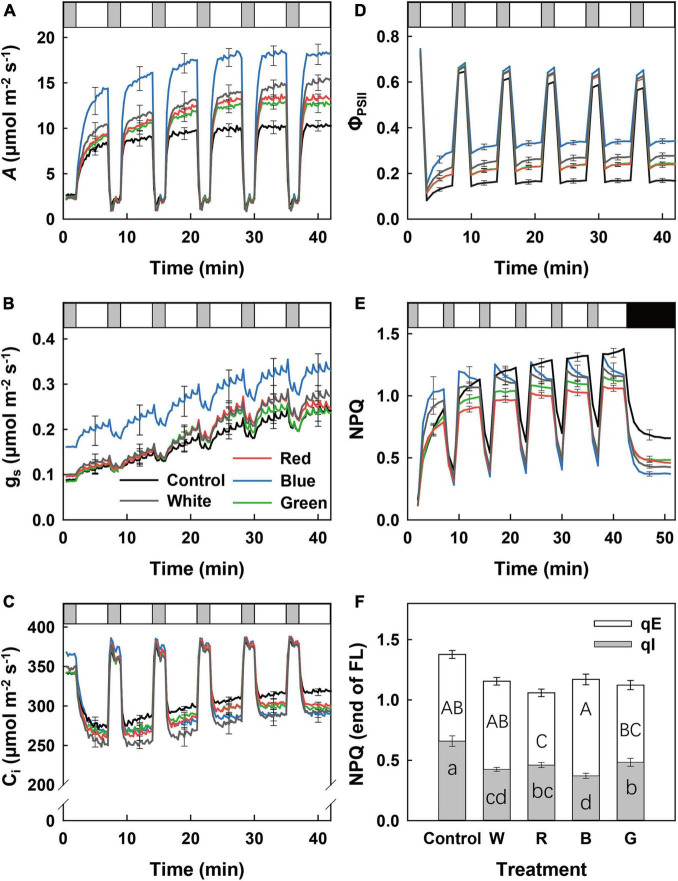
Dynamic leaf photosynthetic traits of cucumber plants grown under different light treatments in response to fluctuating light (FL) intensity. Time courses of net photosynthesis rate (*A*; **A**), stomatal conductance (g_*s*_; **B**), leaf internal CO_2_ partial pressure (C_*i*_; **C**), photosystem II electron transport efficiency (Φ_*PSII*_; **D**), and non-photochemical fluorescence quenching (NPQ; **E**) when a leaf adapted to low irradiance (50 μmol m^–2^ s^–1^) was exposed to FL between 2 min of low light (LL) (50 μmol m^–2^ s^–1^) and 5 min of high light (HL) intensity (1,000 μmol m^–2^ s^–1^) for 42 min (five repeated cycles). NPQ dark relaxation was recorded for another 10 min after FL in panel **(E)**. LL, HL, and darkness are visualized as gray, white, and black bars, respectively. Energy and zeaxanthin-depended quenching (qE) and photoinhibitory quenching (qI) at the transition from HL to darkness are shown in panel **(F)**. Control (C, shade solar light), white (W), red (R), blue (B), and green (G). Mean value ± SEM of three experimental batches are shown (*n* = 3), with three replicate plants per experimental batch. Different letters in panel **(F)** indicate significant treatment effects on qE (capital letter) and qI (lowercase letter), respectively.

When leaves initially adapted to LL (50 μmol m^–2^ s^–1^) were exposed to a series of lightflecks [5 min HL (1,000 μmol m^–2^ s^–1^), interspersed with 2 min LL], *A* increased gradually in all treatments during HL phases (Figure 2A). At any time during fluctuating light (FL), B-grown leaves displayed the highest *A*, followed by W, R, G, and control leaves (Figure 2A). Not surprisingly, integrated *A* during the first 300 s of HL (*A*_300_) was 35–65% higher in B-grown leaves compared to values from other treatments, which did not differ from one another (Supplementary Table 1). Leaves that grew under B also displayed significantly higher g_*s*_ during FL compared with all other leaves (Figure 2B). However, the rate of photosynthetic induction was similar in all treatments, as shown by similar photosynthetic induction states at 60 and 300 s (IS_60_ and IS_300_) after a LL to HL transition (Supplementary Table 1). Control leaves displayed a higher C_*i*_ during HL compared to leaves in other treatments (Figure 2C). A high C_*i*_ along with low *A* (Figure 2A) indicates that photosynthetic limitation in control leaves was mainly due to biochemical rather than stomatal limitation.

At any moment during FL, B leaves showed the highest values for Φ_*PSII*_, followed by W, R, G, and control leaves (Figure 2D). During the initial LL to HL transition, NPQ increased gradually within 5 min in all treatments, with the highest value in B-grown leaves (Figure 2E). However, during the following LL–HL transitions, NPQ showed different patterns among treatments: in B- and W-grown leaves, NPQ increased toward a peak value and then relaxed toward a lower value (Figure 2E). In R- and G-grown leaves, NPQ quickly reached a plateau and did not decrease during HL. Finally, in control leaves, NPQ increased continuously during HL, resulting in a substantially higher value compared with the other treatments (Figure 2E). When light intensity decreased from HL to LL or darkness, NPQ relaxed to a lesser extent in control leaves than in the four supplemental light treatments, whereas B-grown leaves showed the fastest NPQ relaxation rate (Figure 2E). At the transition point from FL to darkness, B-grown leaves showed the highest qE and lowest qI, whereas control leaves showed the highest qI (Figure 2F).

### Quantum Efficiency of Photosystem II in Response to High Light Intensity

**FIGURE 3:**
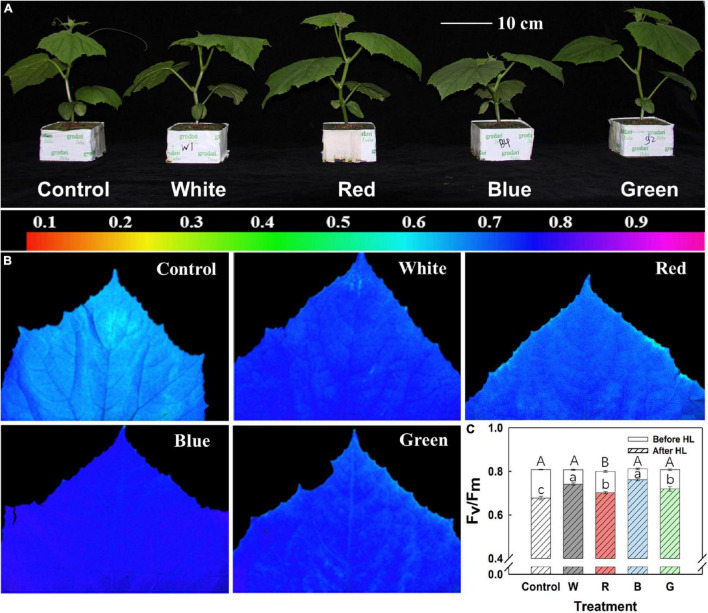
Plant morphology and photoinhibition. **(A)** Representative images showing cucumber plants grown for 10 days under different supplemental light spectra: Control (C, shade solar light), white (W), red (R), blue (B), and green (G). **(B)** Representative false color images of maximum quantum efficiency of photosystem II photochemistry (F_*v*_/F_*m*_) distribution after high light (HL, 30 min, 1,100 μmol m^–2^ s^–1^) treatment, including color scale. **(C)** Averaged F_*v*_/F_*m*_ before and after HL treatment. Mean values ± SEM of four biological replicates in one experimental batch are shown (*n* = 4). Different letters in panel **(C)** indicate a significant treatment effect on F_*v*_/F_*m*_ before (capital letter) and after (lowercase letter) HL, respectively (*p* < 0.05).

F_*v*_/F_*m*_ before and after HL was measured in one batch of the experiment. Before exposure to HL, F_*v*_/F_*m*_ in leaves of all treatments was > 0.8, with a slightly lower value (0.80) in R (Figure 3C). After leaves were exposed to HL (1,100 μmol m^–2^ s^–1^) for 30 min, differences in F_*v*_/F_*m*_ started to show (Figure 3B): B leaves showed the highest F_*v*_/F_*m*_ (∼0.76, 6% decrease from initial value), and control leaves showed the lowest F_*v*_/F_*m*_ (∼0.68, 16% decrease from initial value), whereas W, G, and R leaves showed intermediate drops in F_*v*_/F_*m*_ (Figure 3C). Besides, B leaves showed more homogeneity in F_*v*_/F_*m*_ distribution after HL exposure compared to leaves in other treatments (Figure 3B).

### Plant Growth and Morphology

Plant morphology was strongly affected by the growth light spectrum (Figure 3A). Stems of B-grown plants were the shortest (Table 5). Leaf number, fresh weight, and dry weight of leaves, stems, and whole shoots were similarly increased under all supplemental light treatments (Table 5). Leaf area was increased by 78–94% in W-, R-, and G-grown plants compared with control, whereas in B-grown plants it was only increased by 43% (Table 5). Compared to other treatments, B-grown plants had a tendency to partition more biomass to leaves at the cost of stem biomass (Table 5).

**TABLE 5:** Growth and morphological traits of cucumber plants grown under different supplemental light spectra.

Parameter	Light quality					*p*-value
	Control	White	Red	Blue	Green	
**Leaf**						
Leaf number (>5 cm)	3.70.2b	4.40.5a	4.50.5a	4.30.5a	4.40.5a	0.005
Leaf area (cm^2^ plant^–1^)	31434c	589105a	610125a	45072b	55987*ab*	0.002
Fresh weight (g plant^–1^)	4.40.3b	9.61.5a	9.61.7a	8.11.1a	8.51.0a	<0.001
Dry weight (g plant^–1^)	0.400.04b	1.030.17a	1.020.17a	0.880.11a	0.890.12a	<0.001
**Stem**						
Stem length (cm)	23.02.6*ab*	24.95.9a	28.07.8a	16.62.7b	30.26.2a	0.022
Fresh weight (g plant^–1^)	3.50.3b	7.02.0a	7.32.2a	4.91.2*ab*	7.21.5a	0.023
Dry weight (g plant^–1^)	0.120.01c	0.270.07*ab*	0.290.08a	0.190.04*bc*	0.270.05*ab*	0.014
**Shoot**						
Fresh weight (g plant^–1^)	8.90.7b	19.64.3a	19.94.6a	15.22.9a	18.22.9a	0.005
Dry weight (g plant^–1^)	0.560.05b	1.420.26a	1.420.28a	1.160.16a	1.260.17a	<0.001
DMC (%)	6.260.30c	7.400.36*ab*	7.320.29*ab*	7.810.53a	7.030.34b	<0.001
DMP_leaf (%)	71.11.2c	73.91.9*ab*	72.82.4*bc*	76.41.5a	71.01.6c	0.004
DMP_stem (%)	22.30.9a	18.51.6c	19.82.0*bc*	16.01.0d	21.61.5*ab*	<0.001

*Control (C, shade solar light). DMC, dry mass content; DMP, dry mass partitioning. Mean value ± SEM of four experimental batches is shown (n = 4), with six replicate plants per experimental batch. The p-values of treatment effect are shown, and different letters indicate significant treatment effect.*

### Photosynthesis and Plant Growth After Exposure to Full Sunlight

**FIGURE 4:**
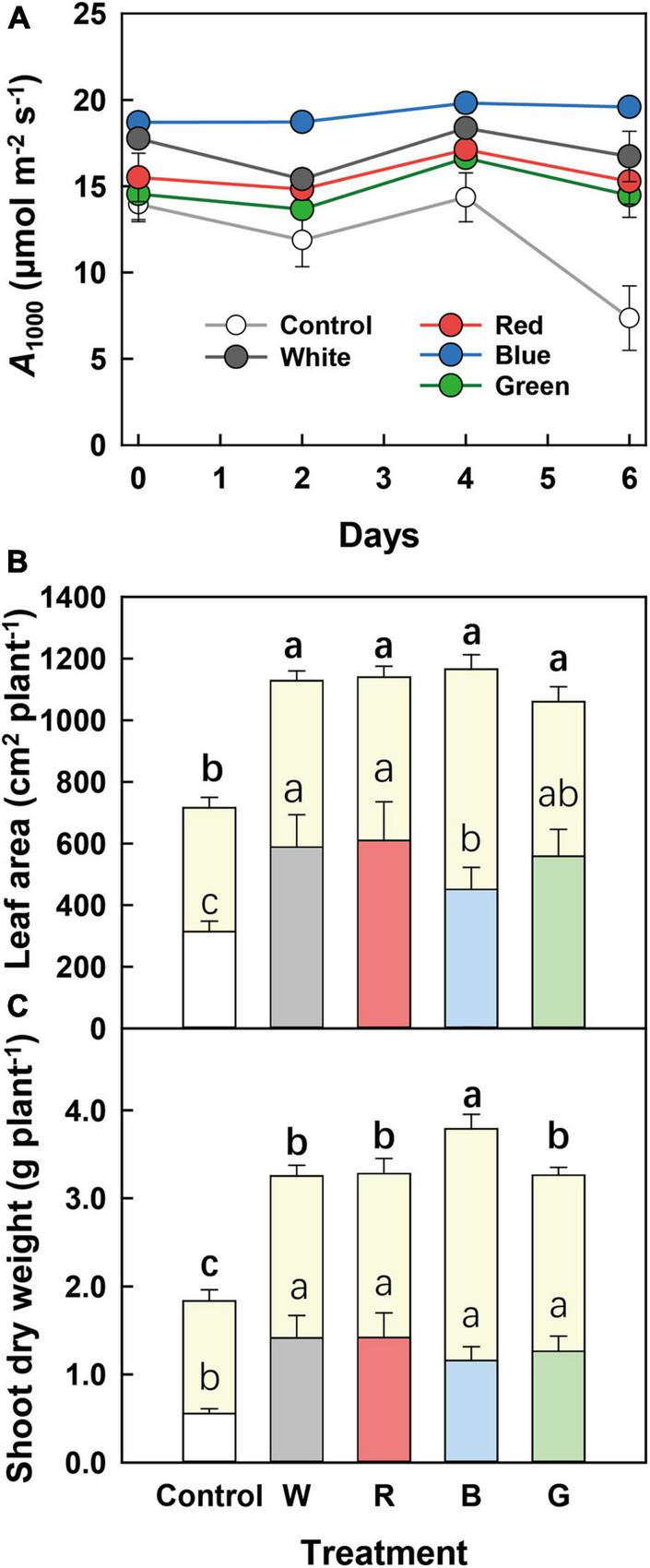
Plant growth and photosynthesis after plant exposure to full sunlight for 1 week. **(A)** Time course of the steady-state net leaf photosynthesis rate at PPFD of 1,000 μmol m^–2^ s^–1^ (*A*_1000_) during 6 days of full solar light exposure. **(B)** Leaf area and **(C)** shoot dry weight of cucumber plants before and after 7 days of full solar light exposure, indicated by the lower and upper part of columns, respectively. Before full solar light exposure, cucumber plants were grown for 10 days under different supplemental light spectra: Control (C, shade solar light), white (W), red (R), blue (B), and green (G). Mean values ± SEM of three to five biological replicates in one experimental batch are shown (*n* = 3–5). Different letters indicate a significant treatment effect (*p* < 0.05).

In the last batch of the experiment, plants from all treatments were transferred to full sunlight for 1 week. *A* at 1,000 μmol m^–2^ s^–1^ PPFD (*A*_1000_; measured every 2 days) was the highest and most constant in B-grown leaves (Figure 4A). Leaves from all other treatments tended to show a larger temporal variability of *A*_1000_, until, on day 6, *A*_1000_ in control leaves displayed a major drop to approximately half of its initial value (Figure 4A). After a week of growth under full sunlight, total leaf area was similar among supplemental light treatments (Figure 4B), though before full solar light exposure B-grown plants had smaller leaf area compared to plants grown under G, R, and W (Table 5). In addition, despite similar leaf and shoot biomass among G-, R-, W-, and B-grown plants before full sunlight exposure (Table 5), a week of full solar light exposure resulted in the highest leaf and shoot biomass in B-grown plants among the treatments (Figure 4C and Supplementary Table 2). This revealed a higher growth rate in B-grown plants during full solar light exposure.

## Discussion

We investigated how acclimation to different supplemental light spectra in the greenhouse with low-intensity sunlight as background affects the plant’s capacity to cope with high and variable light intensity. Our focus was on both photosynthetic and photoprotective capacity, and their kinetics upon light intensity fluctuations. We found that while any supplemental light prepared plants for subsequent HL intensity exposure to some extent (compared to a control without any supplemental light), especially blue light prepared leaves for high solar light exposure best. Thus, leaves grown under supplemental blue light had a higher photosynthetic and photoprotective capacity (compared to other supplemental light spectra) and were able to confer superior plant growth upon transition to higher and more variable solar light.

### Acclimation to Blue Light in Low Solar Light Confers Faster Growth Under High Solar Light

Whole-plant CO_2_ fixation depends on both leaf photosynthetic rate and plant morphology (light interception; Zhu et al., 2010). A high fraction of blue light often led to a compact plant morphology (Hernández and Kubota, 2016; Izzo et al., 2021), and this was characterized by smaller leaf area and shorter stem length compared with other supplemental light treatments in this study (Table 5 and Figure 3A). A similar phenotype was also observed in growth chamber-grown cucumbers (Liang et al., 2021). However, acclimation to blue light conferred a faster growth under high solar light (Figure 4) though with a morphology unfavorable for canopy light interception. Therefore, we ascribe a faster growth rate in blue-light-acclimated plants to efficient utilization of light energy and less deleterious effects associated with exposure to an HL intensity, rather than morphology, compared to plants acclimated to other spectra. The traits in blue-light-acclimated leaves were related to high photosynthetic and photoprotective capacity and the ability of NPQ to induce and decrease quickly upon light intensity changes, as discussed below.

#### High Photosynthetic Capacity in Blue-Light-Acclimated Plants

Manipulating the light spectrum often impacts photosynthesis. Importantly, the effects of monochromatic supplemental light in the greenhouse are likely different from those in the growth chamber, as the realized light spectrum is affected by a broad, solar background light of variable intensity (Kaiser et al., 2019). Specific photosynthetic acclimatory syndromes under a monochromatic spectrum are unlikely in greenhouses. Instead, treatment differences are likely caused by different proportions of wavebands in a broad spectrum.

Previous studies with fully artificial light showed that leaf photosynthetic capacity increases with increases in the fraction of blue light (0–50%) in tomato (Zhang et al., 2019), lettuce (Wang et al., 2016), and cucumber (Hogewoning et al., 2010); our study confirms and expands on these findings, as we found an increase in *A*_*max*_ between 10 and 76% (Supplementary Figure 6). The increase in *A*_*max*_ scaled very well with chlorophyll and nitrogen contents per unit leaf area, leaf thickness (Figures 1D–F), and Φ_*PSII*_ (Figure 2D). In addition, g_*s*_ roughly scaled with *A*_*max*_ (Figures 1B, 2B), and a higher g_*s*_ under blue-light-acclimated leaves was not due to an increased stomatal density, but a significant increase in stomatal size (Table 4). Acclimation to a given waveband, therefore, resulted in a concerted change of all components related to photosynthesis, be it CO_2_ diffusion (Figure 1B), electron transport (Figure 2D), or carboxylation. At a molecular level, blue light can activate cryptochrome and mediate transcription and expression of genes encoding PSII components, assuring a normal development of the photosynthetic apparatus (Walters, 2005; Kleine et al., 2007; Li et al., 2020). At the same time, blue light has been shown to regulate stomatal development (Kang et al., 2009), facilitating CO_2_ availability to improve photosynthetic capacity. Furthermore, blue light might also trigger specific retrograde signals from the chloroplast to the nucleus in a photoreceptor-independent pathway, which can also play an important role in photosynthetic acclimation (Duan et al., 2020; Gommers, 2020).

#### Improved Photoprotective Performance in Blue-Light-Acclimated Leaves

In this study, leaves acclimated to blue light developed the greatest photoprotective capacity under dynamic light intensity, and under constant HL intensity. This was characterized by a highly induced and relaxed NPQ under dynamic light (Figure 2E) and the highest F_*v*_/F_*m*_ after HL treatment (Figures 3B,C).

Non-photochemical quenching plays a key role in plant fitness and productivity, especially under FL. Consistent with previous studies conducted in growth chambers (Zhang et al., 2019; Duan et al., 2020), our study showed that acclimation to blue light can induce the faster formation of NPQ under HL intensity compared to red-light-acclimated leaves. In addition to this, our study showed a faster NPQ formation and relaxation rate in blue-light-acclimated leaves than R-, G-, and W-acclimated leaves (Figure 2E). Therefore, the fast NPQ relaxation in leaves grown under blue light (Figure 2E) may reduce foregone *A* under FL (Murchie and Ruban, 2020). NPQ comprises several components, which are determined by their induction and relaxation time scales. The fastest, and by far largest (under most circumstances), component is qE (Murchie and Ruban, 2020), which enables rapid adjustment of light-harvesting efficiency to incident light. qE can be induced within 10–200 s after a LL to HL intensity transition (Figure 2E). qE is regulated by ΔpH across the thylakoid membrane, which is sensed by PsbS that confers changes in the light-harvesting complex II, where qE takes place and is modulated by the concentration of zeaxanthin (Bassi and Dall’Osto, 2021). A recent study in rice showed that blue light-induced higher PsbS transcript levels, thereby increasing qE capacity in HL intensity, and rate of NPQ relaxation upon a transfer from HL to LL (Duan et al., 2020). We hypothesize that a faster induction of qE in B (during the 1st LL to HL transition, Figure 2E) in our study may be due to increased PsbS concentrations (Duan et al., 2020), and/or faster transthylakoid ΔpH changes due to more rapid electron and proton transport (suggested by larger initial Φ_*PSII*_, Figure 2D).

An effective method for monitoring photoinhibition is the measurement of F_*v*_/F_*m*_ after light stress (Külheim et al., 2002). Leaves acclimated to a high proportion of blue light (B and W light) had the highest values of F_*v*_/F_*m*_ after HL treatment (Figure 3C), again indicating a larger photoprotective capacity compared to leaves in other treatments. The movement of chloroplasts away from HL intensity is an important photoprotective and adaptive mechanism to prevent or recover from the deleterious effects of photoinhibitory light (Liu et al., 2019). Although blue-light-acclimated leaves possessed the highest content of chlorophylls and the thickest leaves (Table 3 and Supplementary Figure 3), surprisingly they did not possess the highest leaf light absorptance (Table 3). This could be related to blue-light-induced chloroplast movement mediated by activated phototropin, which can reduce leaf light absorptance, and may be another protecting mechanism against excess light in blue-light-acclimated plants (Shinkai et al., 2002).

Altogether, a higher proportion of blue light, higher *A* along with higher NPQ capacity, and faster induction and relaxation of qE can not only decrease excess light but also decrease reactive oxygen species production (Liu et al., 2019).

### Acclimation to Supplemental Light Does Not Affect Photosynthesis Dynamics

Under direct light, plants in the greenhouse often experience large variations in light intensity, which are caused by the shade of construction parts and equipment (Supplementary Figure 7). Time-integrated *A* depends not only on the magnitude of steady-state *A* but also on the rapidity of the *A* response to changes in PPFD. The rate at which *A* responds to FL is usually quantified as the rate of photosynthetic induction after low-to-high PPFD transitions (Kaiser et al., 2018; Tanaka et al., 2019).

Under a series of lightflecks aimed at probing *A* under dynamic light, we found differences between treatments during exposure to HL intensity (Figure 2A) that scaled well with those seen from steady-state light responses of *A* (Figure 1A). However, none of the treatments affected the rate of photosynthetic induction (Supplementary Table 1), agreeing with our previous study showing that in tomatoes, acclimation to different R/B ratios had only minor effects on the rapidity of the *A* under FL (Zhang et al., 2019). Photosynthetic induction rate is mainly determined by (1) Calvin cycle enzyme activities, e.g., Rubisco activation rate and (2) CO_2_ diffusional limitation, e.g., transient stomatal limitation (Taylor et al., 2020; Sakoda et al., 2021). Our results again indicate that manipulating the PAR light spectrum does not change Rubisco activation properties or transient stomatal limitation.

### Limitations of Our Study

In this study, we have provided some insights into how the light spectrum can “prepare” plants for HL intensity exposure. Mostly, we base our conclusions on leaf photosynthetic, photoprotective, and biochemical data that were gathered in several independent experiments (Table 2); these results can thus be viewed as very solid. However, for some other measurements, e.g., the growth analysis after full solar light exposure, they were only conducted once (Table 2); these results are only from pseudo-replications rather than statistical replications. Second, to investigate how acclimation to a given spectrum affects plants growth under HL intensity exposure, young cucumber seedlings were transferred to full solar light for 1 week in this study. Ideally, an extension of the HL treatment, e.g., to several weeks, even to a reproductive growth stage (e.g., the fruit yield), will provide a more complete picture as to how long-lasting the effects described here are. Third, strictly speaking, the phrase “full solar light” is not precise: in greenhouses, not only the intensity of solar light is decreased, but also UV radiation is considerably reduced, compared to full solar light in the open field. Therefore, the degree of HL stress after exposure to “full solar light” in the greenhouse is rather mild, compared to that impacting plants in the open field. However, still, it can be hypothesized that plants acclimated to blue light would show a growth advantage when transferred to the open field.

## Conclusion

Our study shows that blue supplemental light in the background of LL can “prepare” plants to develop a high photosynthetic and photoprotective capacity, which subsequently can improve plant growth under full solar light. Although the rate of photosynthetic induction cannot be manipulated by acclimation to supplemental light, maximum leaf photosynthetic capacity and a highly flexible NPQ can be achieved; this may improve light use efficiency and diminish photoinhibition under full solar light exposure, which means both high and highly variable light intensities. Our results may help to bridge the gap between the establishment of young seedlings under different spectra and plant performance after transfer to the open field or greenhouse.

## Data Availability Statement

The original contributions presented in the study are included in the article/Supplementary Material, further inquiries can be directed to the corresponding author.

## Author Contributions

CK and YZ performed the experimental work and drafted the manuscript. RC and QY managed the project. EK revised the manuscript. TL conceived and supervised the study. All authors have read and agreed to the final version of the manuscript.

## Conflict of Interest

The authors declare that the research was conducted in the absence of any commercial or financial relationships that could be construed as a potential conflict of interest.

## Publisher’s Note

All claims expressed in this article are solely those of the authors and do not necessarily represent those of their affiliated organizations, or those of the publisher, the editors and the reviewers. Any product that may be evaluated in this article, or claim that may be made by its manufacturer, is not guaranteed or endorsed by the publisher.

## Abbreviations

*A*: net CO_2_ assimilation rate
*A*_*i*_: steady-state net photosynthesis rate at 50 μ mol m^–2^ s^–1^ photosynthetic photon flux density
*A*_*f*_: steady-state net photosynthesis rate at 1,000 μ mol m^–2^ s^–1^ photosynthetic photon flux density
*A*_*max*_: maximum net photosynthesis rate
α: apparent quantum yield
*A*_300_: integrated *A* during the first 300 s of high light
Ab, Rf, Tr: leaf absorptance, reflectance, transmittance
C: total leaf carbon content
Chl *a* + *b*: total chlorophyll *a* and *b* contents
Chl *a:b*: chlorophyll *a* and *b* ratio
C_*i*_: substomatal cavity CO_2_ partial pressure
DMC: dry mass content
DMP: dry mass partitioning
HL: high light
LL: low light
FL: fluctuating light
F_*s*_: chlorophyll fluorescence yield under light
F_*m*_ ′: maximum chlorophyll fluorescence under light
F_0_: minimal chlorophyll fluorescence under dark
F_*m*_: maximal chlorophyll fluorescence under dark
F_*m*_ ″: maximum fluorescence after 10 min of dark relaxation
F_*v*_/F_*m*_: maximum photosystem II (PSII) efficiency
g_*s*_: stomatal conductance
gs_*i*_: steady-state stomatal conductance at 50 μ mol m^–2^ s^–1^ photosynthetic photon flux density
gs_*f*_: stomatal conductance after 42 min of fluctuating light
IS_60_: photosynthetic induction state at 60 s
IS_300_: photosynthetic induction state at 300 s
LED: light-emitting diode
LMA: leaf mass area
N: total leaf nitrogen
NPQ: non-photochemical quenching
PPFD: photosynthetic photon flux density
PSII: photosystem II
Φ_*PSII*_: photosystem II operating efficiency
qE: energy-dependent quenching
qI: photoinhibitory quenching
R_*dark*_: dark respiration rate
SSL: shade solar light
VPD_*leaf–air*_: leaf-to-air vapor pressure deficit.
