# Supplementary material for: Acclimating Cucumber Plants to Blue Supplemental Light Promotes Growth in Full Sunlight
Source: Front Plant Sci. 2021 Nov 29;12:782465. doi: 10.3389/fpls.2021.782465 (PMC8668241; doi:10.3389/fpls.2021.782465)
Supplement: Supplementary file 1 [file Data_Sheet_1.docx]

**Supplemental information:**

**
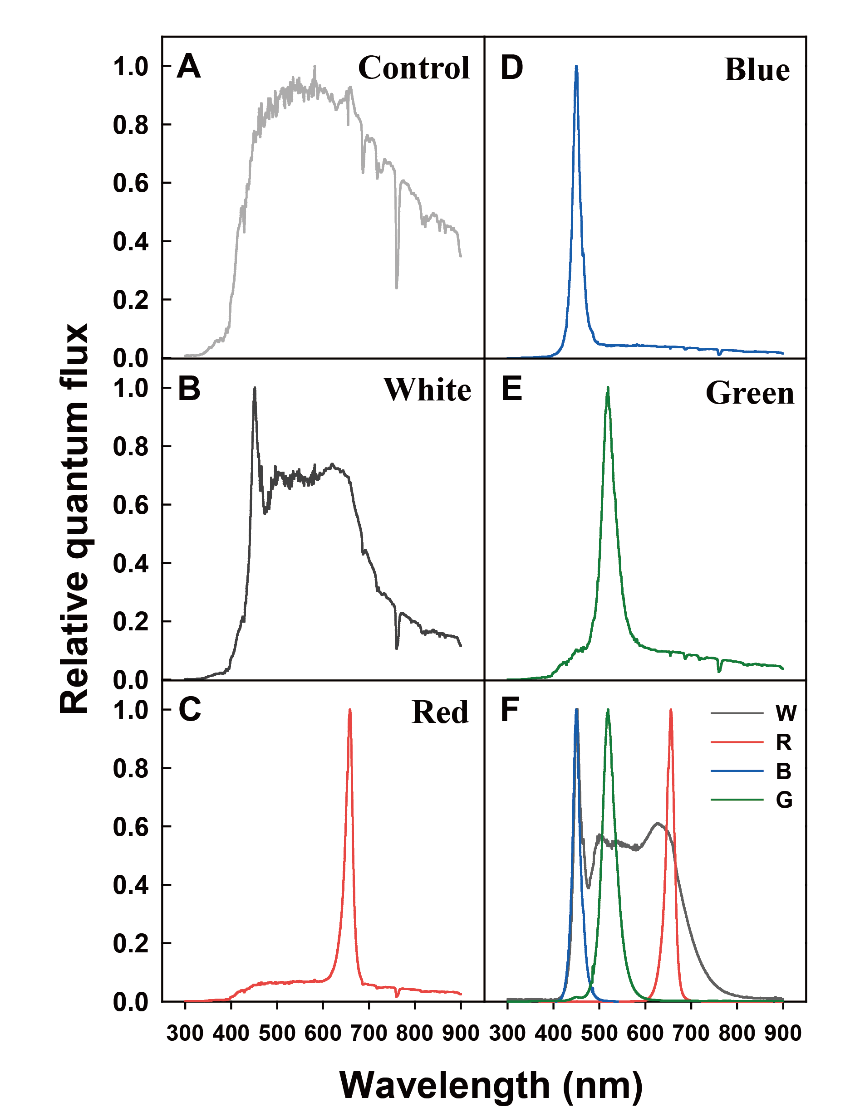
**

**Figure S1.** Relative spectra in the five light treatments: **(A)** Control (C, shade solar light); **(B)** White (W, C+ supplementary white light); **(C)** Red (R, C + supplementary red light); **(D)** Blue (B, C + supplementary blue light); **(E)** Green (G, C + supplementary green light). **A-E**, Above light spectra were measured at noon in a clear day. **(F)** Spectra of the four supplementary LEDs.


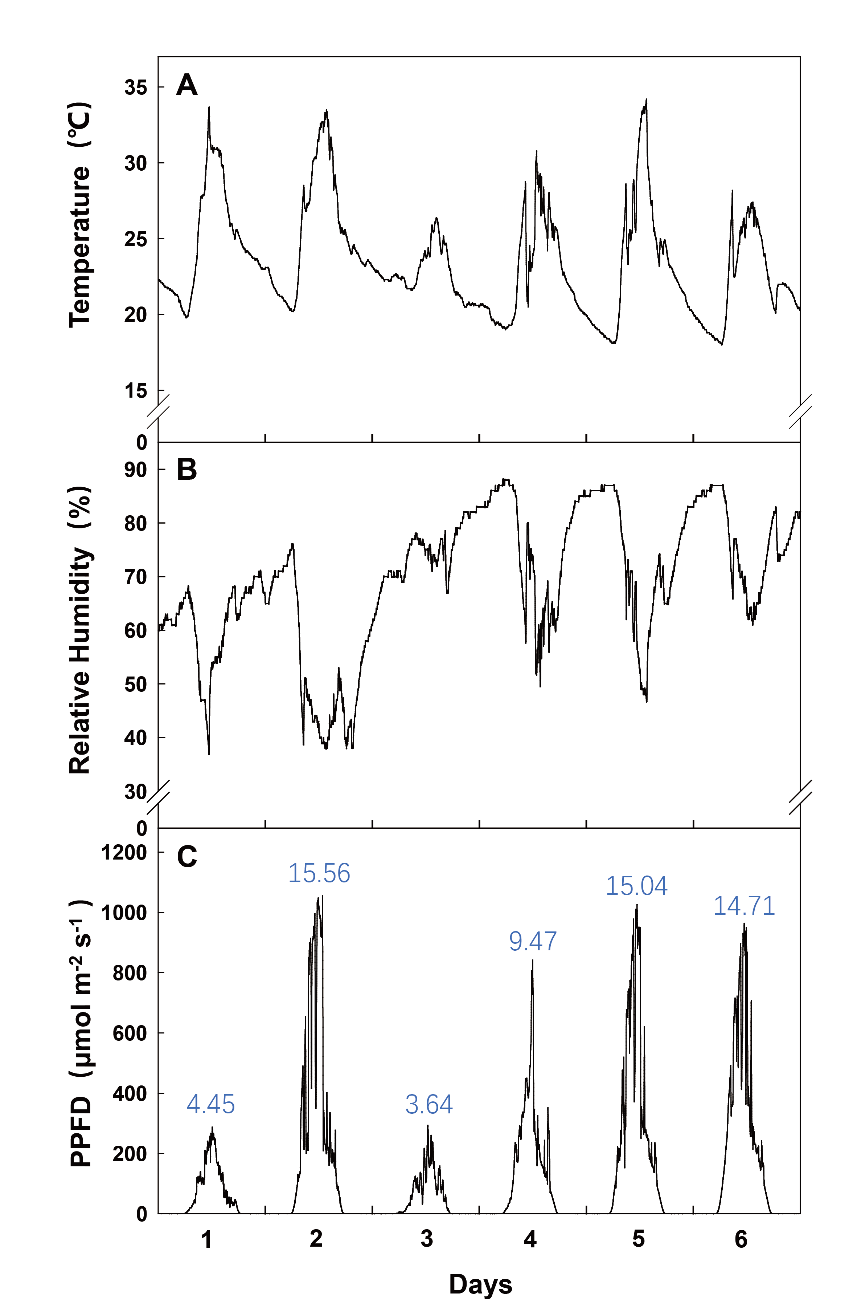


**Figure S2.** Growth conditions during six days of full solar light treatment in the fourth experiment. Numbers in panel C indicate daily light integrals (mol m^-2^) per day.


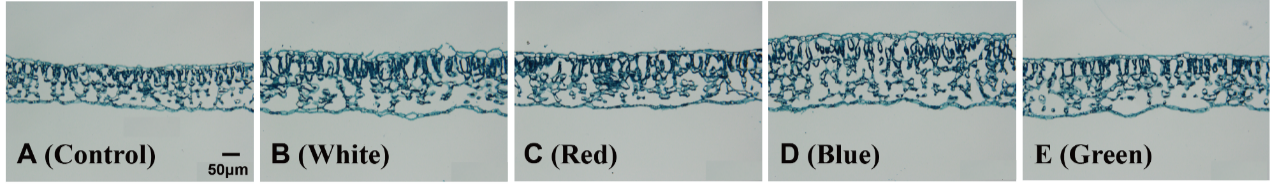


**Figure S3.** Representative images of leaf cross section in cucumber leaves. Cucumber plants were grown for 10 days under different supplementary light spectra: **(A)** Control [C, shade solar light (SSL)]; **(B)** White (W, SSL + supplementary white light); **(C)** Red (R, SSL + supplementary red light); **(D)** Blue (B, SSL + supplementary blue light); **(E)** Green (G, SSL + supplementary green light).


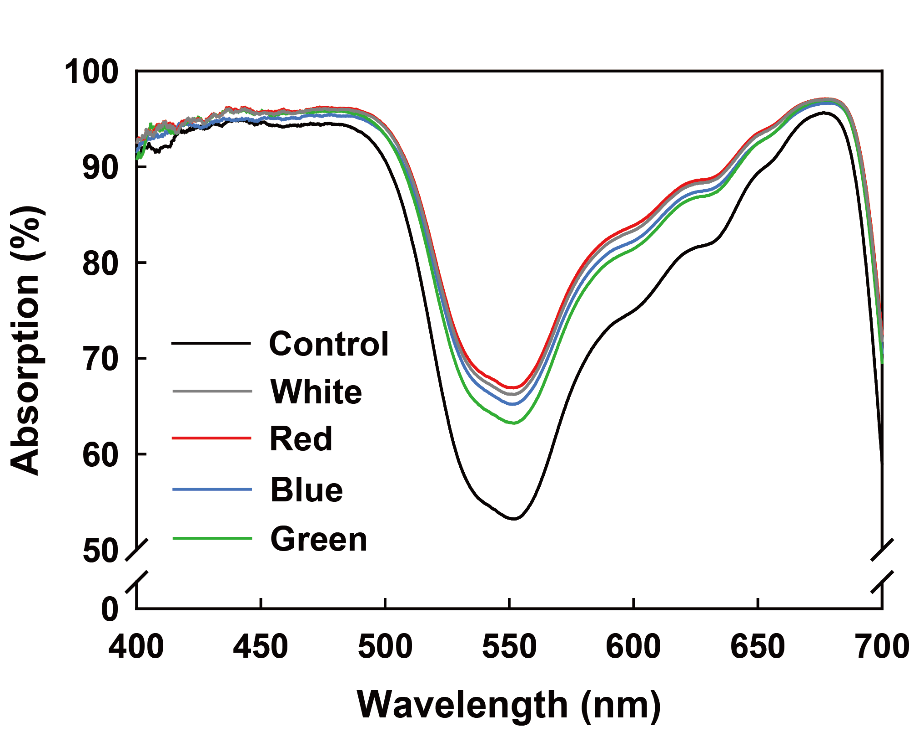


**Figure S4.** Leaf light absorption of cucumber leaves. Cucumber plants were grown for 10 days under different supplementary light spectra: Control [C, shade solar light (SSL)]; White (W, SSL + supplementary white light); Red (R, SSL + supplementary red light); Blue (B, SSL + supplementary blue light) and Green (G, SSL + supplementary green light). Data represent mean value of six biological replicates in one experimental batch (n=6).


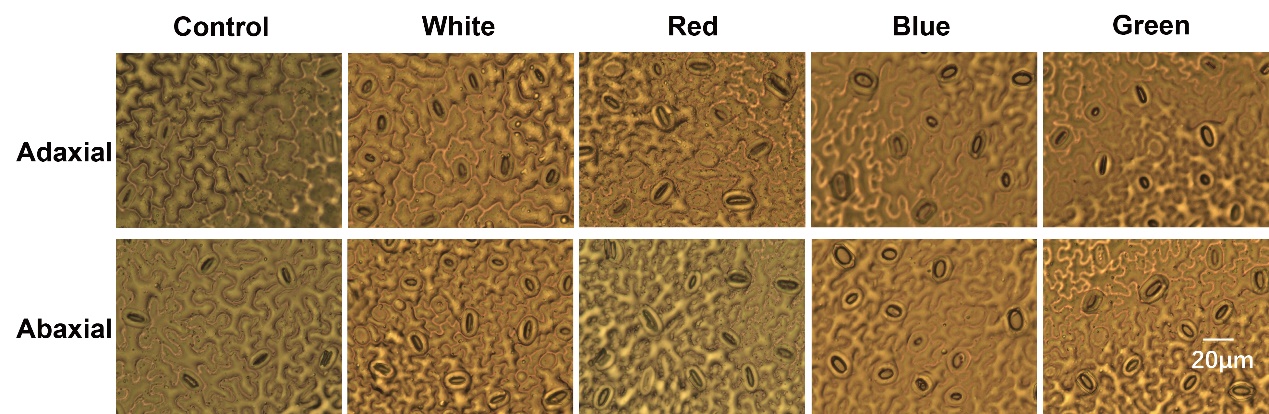


**Figure S5.** Representative images of stomatal traits in cucumber leaves. Cucumber plants were grown for 10 days under different supplementary light spectra: Control, shade solar light (SSL); White, SSL + supplementary white light; Red, SSL + supplementary red light; Blue, SSL + supplementary blue light; Green, SSL + supplementary green light.


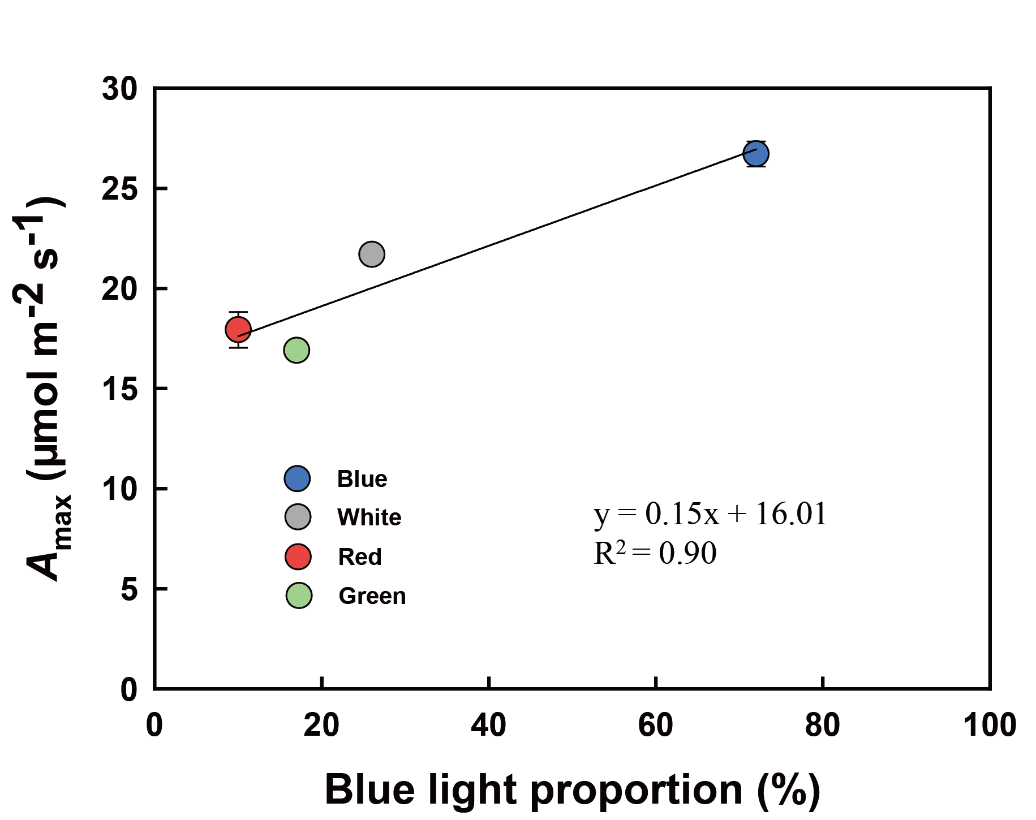
**Figure S6.** The effect of blue light proportion during growth on the photosynthetic capacity (*A*max) of cucumber leaves. Blue light proportion was calculated using an average DLI across four batches of experiments. Mean value ± SEM of 3 experimental batches is shown (n=3), with 4-6 replicate plants per experimental batch.


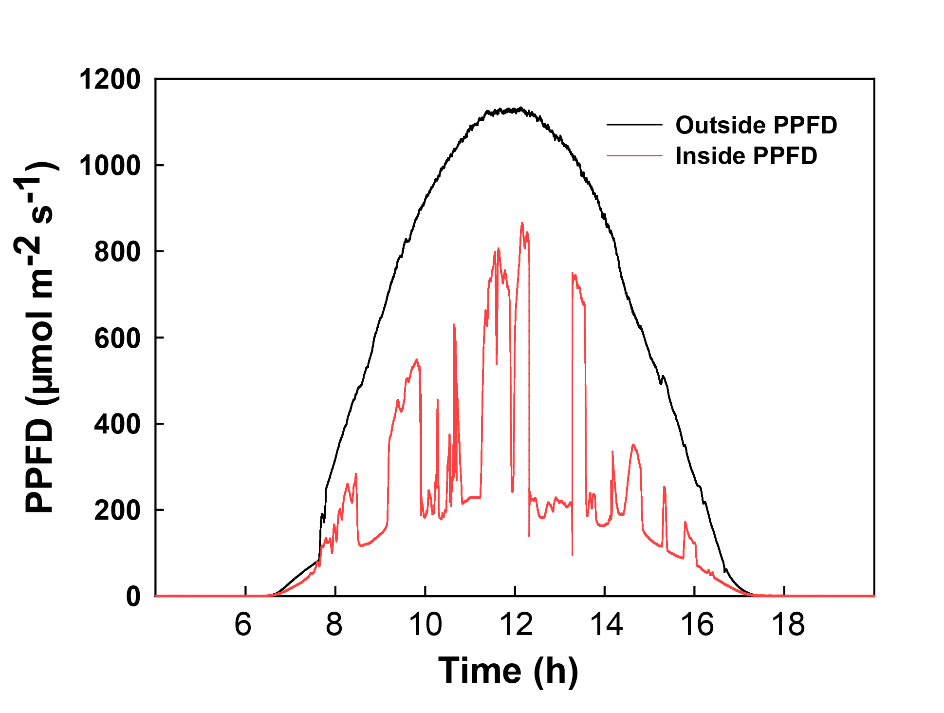


**Figure S7.** Photosynthetic photon flux density (PPFD) in- (above the canopy) and outside the greenhouse (Beijing, China, 40°N, 116°E) on a typical clear day (Nov 1^st^, 2020), as measured by a point sensor.

| **Table S1.** Parameters characterizing dynamic photosynthesis in cucumber leaves in response to fluctuating light (FL). | | | | | | |
| --- | --- | --- | --- | --- | --- | --- |
|  | Light quality | | | | | *P*-value |
|  | Control | White | Red | Blue | Green |  |
| *A*_i_ (μmol m^-2^ s^-1^) | 2.57±0.14 | 2.22±0.14 | 2.19±0.13 | 2.25±0.21 | 2.36±0.16 | 0.081 |
| *A*_f_ (μmol m^-2^ s^-1^) | 10.3±0.6 d | 15.3±0.8 b | 13.4±0.5 c | 18.2±1.1 a | 12.7±0.8 c | <0.001 |
| *A*_300_ (μmol m^-2^) | 2196±118 b | 2696±297 b | 2415±162 b | 3628±375 a | 2373±167 b | 0.001 |
| IS_60_ (%) | 44.2±3.1 | 36.3±4.4 | 37.7±4.6 | 43.6±3.5 | 38.0±3.7 | 0.416 |
| IS_300_ (%) | 71.2±2.3 | 61.3±7.7 | 60.8±6.2 | 74.3±5.3 | 63.9±5.3 | 0.281 |
| gs_i_ (mol m^-2^ s^-1^) | 0.09±0.01 b | 0.10±0.01 b | 0.10±0.01 b | 0.16±0.02 a | 0.09±0.01 b | 0.007 |
| gs_f_ (mol m^-2^ s^-1^) | 0.24±0.02 | 0.28±0.03 | 0.25±0.01 | 0.34±0.04 | 0.24±0.03 | 0.132 |
| Note: Control, shade solar light (SSL); White, SSL + supplementary white light; Red, SSL + supplementary red light; Blue, SSL + supplementary blue light; Green, SSL + supplementary green light. *A*_i_ and *A*_f_, steady-state net photosynthesis rate at 50 and 1000 μmol m^-2^ s^-1^ photosynthetic photon flux density (PPFD), respectively; gs_i_, steady-state stomatal conductance at 50 μmol m^-2^ s^-1^ PPFD; gs_f_, stomatal conductance after 42 min of FL; *A*_300_, integrated *A* during the first 300 s of high light (HL, 1000 μmol m^-2^ s^-1^ PPFD); IS_60_ and IS_300_, the photosynthetic induction state at 60 s and 300 s of HL. Mean value ± *SEM* of three experimental batches is shown (n=3), with 3 replicate plants per experimental batch. *P*-value of treatment effect are shown and different letters indicate significant treatment effect. | | | | | | |

| **Table S2.** Growth and morphological traits of cucumber plants after one week of full solar light exposure. | | | | | | |
| --- | --- | --- | --- | --- | --- | --- |
| Parameter | Light quality | | | | | *P*-value |
|  | Control | White | Red | Blue | Green |  |
| ***leaf*** |  |  |  |  |  |  |
| Leaf number (≥5cm) | 5.6±0.2 | 6.0±0.3 | 6.2±0.2 | 6.0±0.0 | 6.4±0.2 | 0.191 |
| Fresh weight (g plant^-1^) | 11.9±0.7 c | 20.9±0.8 b | 21.2±0.9 b | 23.6±1.0 a | 19.8±0.4 b | <0.001 |
| Dry weight (g plant^-1^) | 1.27±0.08 c | 2.29±0.08 b | 2.31±0.11 b | 2.72±0.10 a | 2.30±0.06 b | <0.001 |
| LMA (g cm^-2^) | 17.7±0.4 c | 20.3±0.5 b | 20.2±0.5 b | 23.3±0.3 a | 21.8±0.7 a | <0.001 |
| ***Stem*** |  |  |  |  |  |  |
| Stem length (cm) | 44.6±1.6 | 45.9±2.2 | 46.9±2.2 | 45.8±2.5 | 50.6±1.7 | 0.318 |
| Fresh weight (g plant^-1^) | 9.3±0.6 b | 14.2±0.7 a | 14.3±0.9 a | 15.8±0.8 a | 14.6±0.6 a | <0.001 |
| Dry weight (g plant^-1^) | 0.43±0.03 b | 0.68±0.04 a | 0.69±0.05 a | 0.76±0.05 a | 0.71±0.02 a | <0.001 |
| ***Shoot*** |  |  |  |  |  |  |
| Fresh weight (g plant^-1^) | 24.6±1.6 c | 41.9±1.8 b | 42.0±1.8 ab | 47.0±2.1 a | 40.6±1.1 b | <0.001 |
| DMC (%) | 7.46±0.11 c | 7.76±0.1 b | 7.80±0.11 ab | 8.06±0.09 a | 8.03±0.05 ab | 0.001 |
| DMP_leaf (%) | 69.6±0.6 | 70.6±0.7 | 70.4±0.9 | 71.7±0.6 | 70.6±0.2 | 0.254 |
| DMP_stem (%) | 23.1±0.4 a | 20.9±0.8 bc | 20.9±0.8 bc | 20.1±0.5 c | 21.8±0.2 ab | 0.014 |
| Note: Before full solar light exposre, cucumber plants were grown for 10 days under different supplementary light spectra: Control, shade solar light (SSL); White, SSL + supplementary white light; Red, SSL + supplementary red light; Blue, SSL + supplementary blue light; Green, SSL + supplementary green light. LMA, leaf mass area; DMC, dry mass content; DMP, dry mass partitioning. Mean value ± *SEM* of five biological replicates in one experimental batch is shown (n=5). *P*-value of treatment effects are shown and different letters indicate significant treatment effects. | | | | | | |
